# Development and validation of a simulator for teaching minimally invasive thoracic surgery in Brazil

**DOI:** 10.1590/ACB360508

**Published:** 2021-06-21

**Authors:** Francisco Martins, Luiz Gonzaga de Moura, Hermano Alexandre Lima Rocha, Josué Viana de Castro, Alexandre Marcelo Rodrigues Lima, Rafael Pontes de Siqueira, Daniela Lima Chow Castillo, Régis Luiz Sabiá de Moura, Acrísio Sales Valente

**Affiliations:** 1 MD, MSc. Service of Thoracic Surgery – Hospital Dr. Carlos Alberto Studart Gomes – Fortaleza (CE), Brazil.; 2 Assistant Professor. Department of Surgery - Centro Universitário Unichristus - Fortaleza (CE), Brazil.; 3 PhD. Fellow Postdoctorate. Department of Global Health and Population - Harvard T. H. Chan School of Public Health - Boston, Massachusetts, USA.; 4 Assistant Professor. Department of Surgery – Universidade de Fortaleza – Fortaleza (CE), Brazil.; 5 MD, MSc. Service of Thoracic Surgery – Hospital Dr. Carlos Alberto Studart Gomes – Fortaleza (CE), Brazil.; 6 MD. Hospital Instituto Dr. José Frota – Fortaleza (CE), Brazil.; 7 MD, MSc. Hospital Instituto Dr. José Frota – Fortaleza (CE), Brazil.; 8 MD, MSc. Senior Engineer at RS Soluções Médicas – Fortaleza (CE), Brazil.

**Keywords:** Thoracic Surgery, Video-Assisted, Education, Medical

## Abstract

**Purpose:**

To develop and validate a chest cavity simulator for teaching video-assited thoracic surgery (VATS).

**Methods:**

The first phase of the study consisted of developing a chest cavity simulator. A quasi-experimental study was performed in the second phase, and 25 surgeons and residents participated in a three-stage pulmonary suture experiment. The videos were recorded and timed. Generalized linear regression models for repeated measures were used to analyze the outcome change over time.

**Results:**

The chest cavity simulator consists of a console simulating the left hemithorax. Among the participants, 96% rated the design, visual aspect, positioning ergonomics, and triangulation of the portals as very good or excellent (face validity). There was a decrease in suturing time in step 1 from 435.7 ± 105 to 355.6 ± 76.8 seconds compared to step 3 (p = 0.001). The evaluation of the simulation effectiveness and performance (content validity) was rated as very good or excellent by 96% ofparticipants. The most experienced surgeon showed significant reduction in procedure time (p = 0.021) (construct validity).

**Conclusions:**

The thoracic cavity simulator is realistic, showing content and construct validity, and can be used in VATS training. The simulation model allowed skill gain in the endoscopic suture.

## Introduction

Video-assisted thoracic surgery (VATS) represented a significant advance in thoracic surgery in the second half of the last century, due to advantages such as shorter length of stay in hospital, reduced pain, reduced morbidity and faster return to everyday activities^1^–^3^. A published study on VATS lobectomy with 1,015 resections for the treatment of lung cancer demonstrated that the three-port technique was safe, reduced morbidity and mortality, in addition to being effective in oncological patients^4^.

In the model proposed by Halstead, the skill gain was based on the performance of a large number of procedures in patients^5^. Operating room learning costs are known to be expensive. The simulation training brought skill gains that can be transferred to the operating room, such as performance gain and errors reduction, offering an unlimited number of repetitions and, most importantly, it does not harm the patients during training^6^–^8^. Practicing cannot be understood as weakness, but as synonymous with responsibility and ethics^9^.

Currently, there are no specific simulators for VATS training in Brazil, and few simulation models are available. Virtual simulators involve high equipment acquisition costs and a small number of procedures available for VATS simulation^10^. Therefore, the development of simulators involving specific and realistic simulation models is needed.

## Methods

### Ethical aspects

The study was analyzed and approved by the Research Ethics Committee of the Centro Universitário Christus (Unichristus) (REC protocol 03129118.2.0000.5049), according to Resolution no. 466/12 of the National Health Council. The research was carried out after approval, and the research participants signed the Free and Informed Consent Form.

An experimental study was carried out in two stages: the chest cavity simulator’s construction, and a pulmonary suture training model.

#### Phase I: Chest cavity simulator

**Figure 1:**
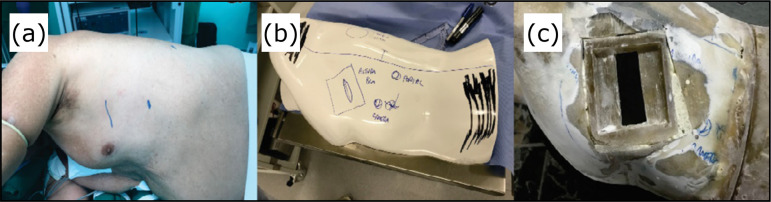
Teaching model. **(a)** Human chest in right lateral decubitus. **(b)** Teaching model simulating the left hemithorax and incisions of a three-port video-assited thoracic surgery. **(c)** Console.

A teaching model was developed based on the human chest in lateral decubitus, and only the left hemithorax was reproduced (Fig. 1), according to frequently used models in simulation^11^.

The thoracic surgeon and an engineer adapted the size of the teaching model and the positioning of the ports, the ideal depth for the artificial lung, in addition to the positioning of the cameras and the monitor.

A 13 × 9-cm base was prepared, combined with an auxiliary thoracotomy (AT), covered with thermoplastic elastomer (TPE) with a central opening measuring 6 × 2.5 cm, allowing access to the cavity.

To offer an ideal image with an appropriate angle, distance and triangulation of the instruments, adjustments were necessary for the ideal positioning of the camera attached to the console.

At the end of this phase, the simulator measured 45 × 28 × 24 cm, with an auxiliary thoracotomy at 5 cm from the camera, 7 cm from the anterior port and 11 cm from the posterior port. The monitor was placed in a posterior position to the teaching model and elevated using a rod for better ergonomics.

An artificial lung was developed with a three-dimensional (3D) printer with a size compatible with the teaching model chest cavity, measuring 18 × 13 × 5 cm. It was used as a template for the lungs manufactured in TPE, with an estimated weight of 250 g.

A sagittal section was cut on the lower part of the teaching model, using telescopic slides for mobility, allowing the placement of any appropriately sized piece.

#### Phase II: Pulmonary suture training model

A pulmonary suture model was performed on a TPE lung piece to evaluate and validate the simulator.

### Study type and population

An experimental and prospective study was carried out, during the XXI Brazilian Congress of Thoracic Surgery, in May 2019, Belo Horizonte, Minas Gerais, Brazil.

### Sampling

Twenty-five surgeons who had already completed their training or residency in Thoracic Surgery were recruited at random. The sample consisted of 22 men and three women.

### Inclusion criterion

Thoracic surgeons and thoracic surgery residents aged between 25 and 75 years old.

### Exclusion criteria

Surgeons who did not complete all the experiment stages due to health conditions, fatigue, or because they decided not to continue up to the final stage were excluded.

Surgeons who did not follow the proposed pulmonary suture model were excluded as well.

### Materials

To carry out the experiment, we used:

- chest cavity simulator;
- TPE lung with elevated incisions measuring 4 cm in length;
- 26-cm Edlo® needle holder, 30-cm MIS DeBakey Wexler® surgery forceps, 40-cm Knot pusher RS Soluções Médicas®;
- 3 polyglactin suture thread, QualTrust Ethicon*®,* measuring a total length of 70 cm, 26-mm ½ needle;
- iPhone XS® digital stopwatch;
- 11’ MacBook Air® computer;
- video camera 700 TVL USB AV endoscopy camera (Zirion®), coupled to a DVR, which in turn was connected to the monitor, and a 5-mm and 30º Striker® optic.

### Intervention description

The participants filled out a structured form, and data were collected on the participants’ training and medical graduation level and their surgical skills.

All participants watched a video demonstrating how to perform the suture with an explanation about the technical details. The participants carried out the experiment in three consecutive steps, and, at the end, they evaluated the simulator and the simulation.

The experiment consisted of performing a pulmonary suture in two planes, with a total length of 4 cm, using the Greek suture technique in the most profound plane and running suture in the superficial plane. All suture steps were timed, starting at the moment the needle holder entered the simulator and stopping when the last knot was completed.

Participants were given feedback at the end of step I, and between steps II and III the feedback was given during the experiment (concurrent feedback), whereas another feedback was given at the end of the steps to improve performance and reduce time and errors.

At the end of the experiment, all participants performed a post-procedure evaluation, and information was collected about the simulator and the possibilities of using it as a teaching model.

Questions were asked about the simulator characteristics and the simulation as stated in the evaluation questionnaire by Moura Júnior. The Likert scale was used to evaluate and score the simulator, considering very bad (number 1)the worst evaluation and excellent (number 5) the best evaluation.

### Evaluation of the pulmonary suture experiment

To compare the skills in progression through the stages, the procedures were timed and recorded, with a total of three per participant and 75 in total. The videos were edited and anonymized by the researcher and speeded up by 30%, and designated as video 1, video 2, up to video 75. In groups of 15 videos, these videos were randomly sent to two experienced VATS surgeons, who, individually, watched all the 75 videos with the total duration of 337.31 minutes.

The sutures were then manually assessed by the tutor using a global rating scale adapted to the procedure as part of the objective structured assessment of technical skill (OSATS)^6^. Another evaluation was carried out through an error scale created by the author, in which a point was assigned to each error made by the participant, and more than one point could be attributed to the same error. The items of the scale were: cross the wire; fray the tissue; do not contemplate all tissue beds; a very loge surgical knot; a remarkably close surgical knot; break the thread; knead the needle; tear the tissue; loose surgical knot base; aerial surgical knot; break the thread; less than three surgical knots; and misuse of the surgical knot depressor.

OSATS 1 evaluation was considered for evaluator 1 and OSATS 2 for evaluator 2. OSATS average was considered as the mean between the measures of evaluators 1 and 2. Error evaluation 1 was considered for evaluator 1 and error evaluation 2 for evaluator 2. Error means comprised the mean of the error measures between evaluators 1 and 2.

### Variables

Epidemiological variables were used to assess the participants’ information, as well as time, progression scale and errors, likewise variables from the evaluation questionnaire regarding the simulator.

### Independent variables

The measured variables comprised the level of training in thoracic surgery by video, VATS lobectomy, lobectomies by thoracotomy, as well as previous experience with endo-sutures.

### Dependent variables

- Total suture time: the total suture time of all study participants in the three stages was timed;
- Overall performance evaluation score: the scores given to each item of the global assessment scale were added up and expressed asvalues;
- Overall score of the error scale: the two evaluators carried out an error scale scoring a point for each repeated error, obtaining a final score comprising all errors.

### Variables of the simulator and simulation evaluation questionnaire

After the three steps, the participants answered the questions related to the chest cavity simulator, expressed on a Likert scale. The scored variables were: visual appearance, simulator design, port distribution, triangulation suitability, positioning ergonomics, image quality, fulcrum effect, technical resource for an assistant surgeon, resource to incorporate technology, performance, and effectiveness.

The participants were asked to evaluate the resistance and resilience of the material used in the simulation.

### Statistical analysis

The descriptive analysis involved the evaluation of the absolute count and frequency for the qualitative variables, and verification of quantitative data normality, using the Shapiro-Wilk test. Also, the variance between groups was verified using Levene’s test. For comparison between groups of data, the χ^2^ test for qualitative variables was used. Student’s *t*-test and the one-way analysis of variance (ANOVA) were applied to compare means/medians of continuous variables according to the distribution of data between groups. Generalized linear regression models for repeated measures were used to analyze the outcome change over time. P < 0.05 was considered significant. All analyses were performed using the software Statistical Package for the Social Sciences (SPSS Statistics®) for MAC OSX, version 23.0 (IBM, United States).

## Results

### Simulator

**Figure 2:**
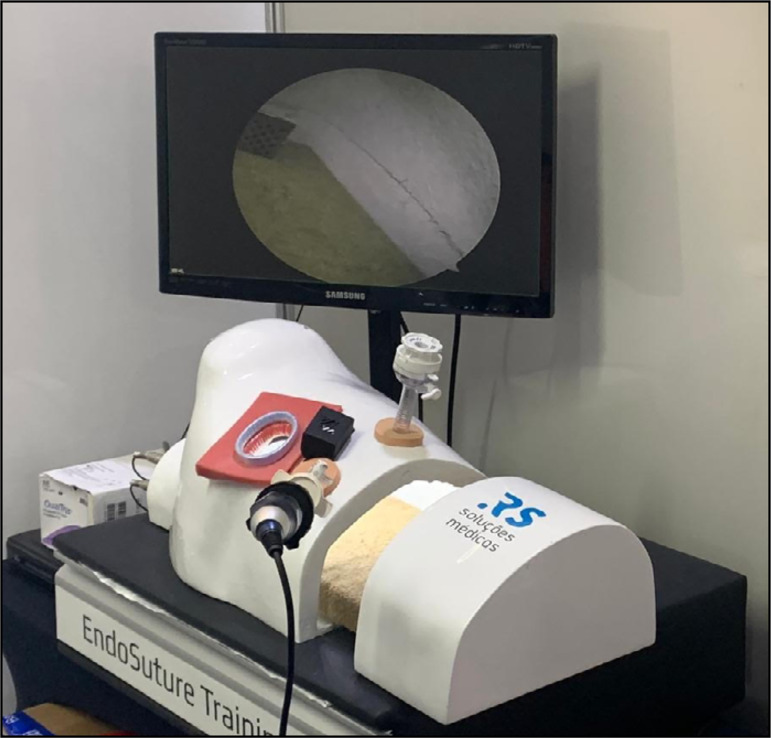
Chest cavity simulator reproducing the left hemithorax.

The cost of the simulator (Fig. 2) was 6,900 BRL (about US$ 1,200) and included the following parts:

- Fiberglass console, reproducing the left hemithorax, with an auxiliary thoracotomy (AT) and two access ports similar to those used in three-port VATS;
- Wooden furniture, with one drawer and covered with leather imitation;
- A 22” LCD Samsung® monitor, with 1,366 × 768 resolution, with a gas piston monitor support;
- SK-c600® fixed camera, with a 720-line resolution, placed on the top of the console, at 5 cm from the AT, with a conventional coupling of optical and video systems;
- Silicone EVA thoracoscopic ports;
- Electrical components such as light-emitting diode (LED) points, control plug, image cable, power supply, and on/off switch.

### Pulmonary suture experiment

#### Sample

The sample consisted of 25 surgeons, predominantly males (88%), with age ranging from 30 to 60 years old (mean of 41.2 ± 8). The time since medical school graduation was 17.8 ± 8.2 years, and they had finished thoracic surgery residency 12.6 ± 9.6 years before. Among the participants, 26.1% reported playing a musical instrument.

Most surgeons were experienced in performing VATS (72%) and using it frequently (mean of 69.2 ± 48.1 procedures in the last 12 months). Among them, 76% said they took video-assisted surgery courses, and 84% had already performed video-assisted pulmonary sutures.

#### Suture time

**Figure 3:**
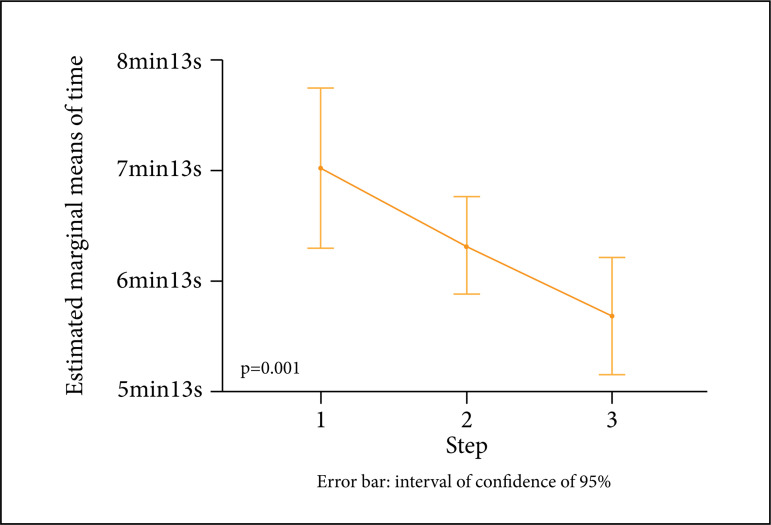
Estimated marginal means of time in relation to the three stages (p < 0.05) (n = 25).

There was significant reduction in the suture time between the three stages, ranging from 435.7 ± 105 to 355.6 ± 76.8 s, with a decrease of more than 1 minute between stages I and III, being statistically significant (Fig. 3).

**Figure 4:**
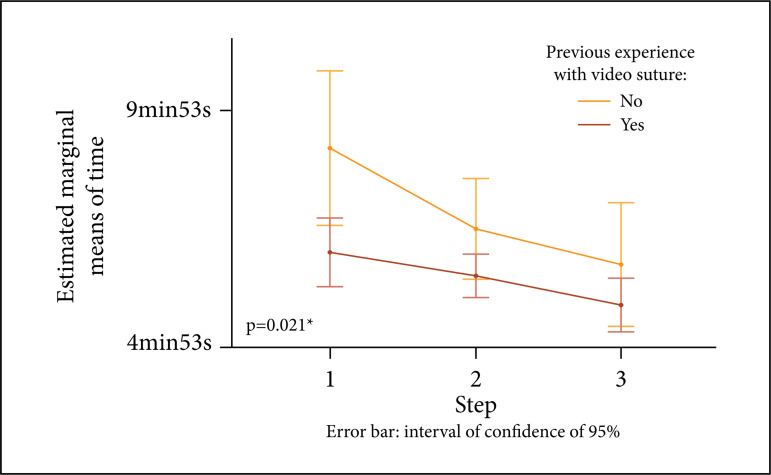
Suture time regarding previous experience with video suture in relation to the three stages (p < 0.05) (n = 25).

The previous participation in any other VATS course was associated with a shorter suture time, but without statistical difference. However, reduction in suture time was observed when comparing the performance of those who had previous experience with video suture to those who did not have any experience, with the difference being statistically significant (Fig. 4).

When analyzing the relationship between suture time and proficiency in VATS lobectomy, using 50 lobectomies as the cutoff point^12^, a shorter suture time was observed for the group with more than 50 lobectomies, with a statistically significant difference for time 3 (p < 0.005).

### Simulator evaluation

None of the simulator assessments were rated as bad or very bad. Among the participants, 96% rated the simulator’s visual appearance, design, and positioning ergonomics as excellent or very good, as well as its performance and effectiveness. One hundred percent of the participants rated port distribution and triangulation adequacy as excellent or very good (Table 1).

**Table 1:** Surgeons’ evaluation of the simulator showing the analyzed parameters (n = 25).

Variables	n (%)
Visual appearance	
Good	1 (4)
Very good	13 (52)
Excellent	11 (44)
Simulator design	
Good	1 (4)
Very good	8 (32)
Excellent	16 (64)
Port distribution	
Very good	8 (32)
Excellent	17 (68)
Triangulation adequacy	
Very good	9 (36)
Excellent	16 (64)
Positioning ergonomics	
Good	1 (4)
Very good	6 (24)
Excellent	18 (72)
Operative field visibility / Image quality	
Good	3 (12)
Very good	7 (28)
Excellent	15 (60)
Resistance and resilience feedback	
Good	1 (4)
Very good	11 (44)
Excellent	13 (52)
Fulcrum effect	
Very good	12 (48)
Excellent	13 (52)
Technical resource for assistant surgeon	
Good	1 (4)
Very good	9 (36)
Excellent	15 (60)
Resource to incorporate technology	
Good	1 (4)
Very good	5 (20)
Excellent	19 (76)
Performance and effectiveness	
Good	1 (4)
Very good	5 (20)
Excellent	19 (76)

#### Video evaluation of the objective structured assessment of technical skill and the error scale

The marginal means of OSATS 1 demonstrated a favorable evolution over the three stages of the experiment, with a progressive increase, but without statistical significance, similar to the one of OSATS 2.

When analyzing the estimated marginal means of errors 1, there was decrease in the average of errors between the first and second stages, with a slight increase from the second to the third stage, without statistical difference. However, when analyzing the estimated marginal means of errors 2 and the graph of errors 1, there was a decrease in the average errors from the first to the second stage, and an increase from the second to the third stage, albeit without any statistical difference between them.

## Discussion

### Simulator

The simulator developed in the present study resembles a human hemithorax and uses VATS positioning in three ports^4^. As it is equipped with an internal camera system, it can be transported and used anywhere, at an affordable cost. In addition, 96% of the research participants rated the simulator’s design shown in this study as very good and excellent, including the visual aspect, positioning ergonomics, and port triangulation. Based on that, the simulator developed herein is a realistic representation of a human chest, together with the positioning and triangulation as seen in VATS, establishing face validity^13^,^14^.

Several realistic simulation models are available, such as black-box simulation, virtual simulators, and simulation using live animals. VATS lobectomy in animals tests the simulation of an upper left lobectomy in pigs^11^. Virtual simulators are expensive, and few models are available for thoracic surgery. Models that use live animals are costly and are usually available for a single use^15^. Although the simulation in live animals is a realistic one, everyday use is difficult and costly, in addition to the ethical problems involved in it.

In the Brazilian market, the available simulators are the same ones used in video-laparoscopy, which are square-shaped and have a triangulation that is not similar to that one used in VATS^16^–^18^ which was recorded and assessed blindly and independently by 2 thoracoscopic experts using a modified version of a validated assessment tool. RESULTS: Interrater reliability was acceptable (Spearman *ρ* = 0.73, P < 0.001. There are few available simulators in the international market, and they require a video monitor, lighting, and video processor equipment similar to those used in surgical centers^19^, which hinders their use and increases the costs.

### Simulation

There was a decrease in the suture time measured during the three stages studied. The decrease can be explained by repeated training in a safe and simulated environment, as already described by Stefandis *et al*.^20^ Moreover, 96% rated the effectiveness and performance of the simulator as very good or excellent when simulating a lung parenchyma suture. The ability to simulate training with an evaluation higher than 80% and involving skill gains demonstrated the content validity of the simulation^20^,^21^

Construct validity is characterized by the ability to differentiate the most experienced from the least experienced surgeons during a simulation^22^,^23^. The simulator developed here demonstrated a construct validity evidenced by the shorter suture time for surgeons with more experience in VATS lobectomies and with previous experience in the endoscopic suture.

The teaching of surgical techniques and surgery simulation gave rise to the need for mechanisms to evaluate the procedure, and OSATS has been widely used in several surgical areas for skill evaluation^20^,^24^ including thoracic surgery^17^. The global rating scale used in the present study, adapted from the OSATS, demonstrated an improvement in continuous assessment from steps 1 to 3, but only the scores assigned by examiner 2 were statistically different. The evaluation of the score on the error scale showed decrease in the number of errors in step 3 in relation to step 1, but without statistical significance. The lower complexity of the procedure can explain this fact.

The present study has limitations related to the small sample size, few suturing steps, and no translational validation, so that the surgeons’ performance in the operating room would have to be evaluated before and after the experiment. It is worth mentioning that translational validation has technical and ethical difficulties towards its performance^25^.

## Conclusion

The chest cavity simulator presented here was face, content, and construction validated. The simulator may also be validated in future research to allow simulation of other tasks. The pulmonary suture simulation model improves surgeon performance in endoscopic suture in the thoracic surgery field.
